# P-1269. Genetic markers of antimicrobial resistance in oropharyngeal commensal Neisseria

**DOI:** 10.1093/ofid/ofaf695.1459

**Published:** 2026-01-11

**Authors:** Huan V Dong, Paul C Adamson, Grace Aldrovandi, Shangxin Yang

**Affiliations:** UCLA, Los Angeles, CA; UCLA School of Medicine, Los Angeles, California; UCLA, Los Angeles, CA; Univeristy of California, Los Angeles, Los Angeles, California, USA, Los Angeles, CA

## Abstract

**Background:**

Non-pathogenic *Neisseria* species are of increasing significance given their potential of harboring antimicrobial resistant (AMR) genes and studies have shown horizontal gene transmission (HGT) can be responsible for antimicrobial resistance in pathogens such as *Neisseria gonorrhoeae*.

**Methods:**

From 5/2022 – 12/2023, oral rinse specimen from men in a HIV pre-exposure prophylaxis (PrEP) program were plated on LBVT-SNR media for culture. Minimum inhibitory concentrations (MIC) to antibitoics were determined using E-tests. Isolates were sequenced using Illumina and analyzed using Geneious Prime software. Species identification resulted from NCBI blasts of *16s* and *rpoB* gene-aligned sequences. AMR genes were identified using Resfinder (4.5.0) from the Center for Genomic Epidemiology.

**Results:**

In total, 48 *Neisseria* isolates were grown from 42 different individuals, with *N. subflava* being the most prevalent species (n=23). Of the 48 isolates, 41 (85%) were resistant to Azithromycin, 3 (6%) to Cefixime, 3 (6%) to Ceftriaxone, and 26 (54%) to doxycycline. The *msr(D)* gene was found to have sensitivity of 31.5%, specificity 85.7%, positive predictive value (PPV) 92.9%, and negative predictive value (NPV) 17.7% for overall Azithromycin resistance, yet sensitivity 83.3%, specificity 88.9%, PPV 71.4%, NPV 94.1% for high-level Azithromycin resistance (MIC ≥ 256 μg/mL). The *blaTEM-1B* gene had sensitivity of 100%, specificity 91.1%, PPV 41.8%, and NPV 100 % for both cefixime and ceftriaxone resistance. Detection of any *tet gene (B/G/M)* had a *sensitivity 46.2%, specificity 81.8%, PPN 74.9%, and NPV 56.4% for doxycycline resistance.*

**Conclusion:**

Prevalence of AMR genes detected in multiple commensal *Neisseria* species showed the potential of HGT to pathogenic *Neisseria* species. Strikingly, 83% of isolates with high-level azithromycin resistance had the *msr(D)* gene. In this study, detection of *msr(D)* had high NPV for high-level resistance and high PPV for overall resistance to Azithromycin. Our study sheds light on the previously underappreciated dynamic of AMR gene reservoir and inter-species transfers in the oropharyngeal microbiome that may play an important role in the rapid increase of multi-drug-resistant *N. gonorrhoeae*.

**Disclosures:**

Shangxin Yang, PhD, D(ABMM), Eurofins | Viracor Clinical Diagnostics: Advisor/Consultant

